# Meniere’s disease: Still a mystery disease with difficult differential diagnosis

**DOI:** 10.4103/0972-2327.78043

**Published:** 2011

**Authors:** A. Vassiliou, P. V. Vlastarakos, P. Maragoudakis, D. Candiloros, T. P. Nikolopoulos

**Affiliations:** National Institute for the Deaf, Athens, Greece; 1 Lister Hospital, East & North Hertfordshire NHS Trust, Stevenage, United Kingdom; 2 Atticon University Hospital, Athens, Greece

**Keywords:** Diagnosis, electrocochleography, glycerol test, Meniere’s disease, vertigo

## Abstract

One hundred and forty-six years after its first description, the differential diagnosis of Meniere’s disease remains very challenging. The aim of the present study is to review the current knowledge on the advantages and disadvantages of the new diagnostic methods for Meniere’s disease. The importance of accurate diagnosis for primary healthcare systems is also discussed. An extensive search of the literature was performed in Medline and other available database sources. Information from electronic links and related books were also included. Controlled clinical studies, prospective cohort studies, retrospective cohort studies, cross-sectional studies, case reports, written guidelines, systematic reviews, and books were selected. The typical clinical triad of symptoms from the vestibular and cochlear systems (recurrent vertigo, fluctuating sensorineural hearing loss and tinnitus) is usually the key for clinical diagnosis. Glycerol dehydration test and electrocochleography are the main diagnostic tests in current practice, while vestibular evoked myogenic potentials may be used in disease staging. Imagine techniques are not specific enough to set alone the diagnosis of Meniere’s disease, although they may be necessary to exclude other pathologies. Recently developed 3D MRI protocols can delineate the perilymphatic/endolymphatic spaces of the inner ear and aid diagnosis. Meniere’s disease is a continuous problem for the patients and affects their quality of life. Taking into account the frequent nature of the disease in certain countries, efforts for reliable diagnosis, prompt referral, and successful management are undoubtedly cost-effective for healthcare systems.

## Introduction

Meniere’s disease is a disease of the inner ear, characterized by the clinical triad of recurrent vertigo, fluctuating sensorineural hearing loss, and tinnitus.[1] The relapsing nature of the disease may significantly affect the patients’ quality of life, especially during periods of acute symptomatology.[2,3] Vertigo mainly influences the physical dimension, while tinnitus and hearing loss influence the psychosocial dimension of patients’ lives.[4]

Clinical symptoms and audiometric tests are the basis for the diagnosis; however, differential diagnosis may be extremely difficult, since most of the findings are subjective and not specific. Misdiagnosis is, therefore, probable, thus highlighting the great need for objective and reliable testing.

The aim of the present study is to review the current knowledge on the advantages and disadvantages of the new diagnostic methods for Meniere’s disease. The importance of accurate diagnosis for primary healthcare systems and the implications of potential misdiagnoses are also discussed.

## Materials and Methods

An extensive search of the literature was performed in Medline and other available database sources, using the key words “Meniere’s disease”, “vertigo”, “tinnitus”, “diagnosis”, “electrocochleography”, and “glycerol test”. The key word “Meniere’s disease” was considered primary and was either combined to the other key words individually, or used in groups of three. In addition, reference lists from the retrieved articles were manually searched. Information from electronic links and related books were also included in the analysis of data.

Four controlled clinical studies, 16 prospective cohort studies, 10 retrospective cohort studies, 1 cross-sectional study, 2 case reports, 1 written guideline, 6 systematic reviews, and 3 books met with the defined criteria and were included in study selection.

## Discussion

### Definition, staging and epidemiology of Meniere’s disease

The term Meniere’s disease is widely used to describe the clinical triad of recurrent rotatory vertigo, fluctuating sensorineural hearing loss and tinnitus. This triad of symptoms was first described by Prosper Meniere in 1861.[1] In addition, the sensation of aural pressure and fullness in the ear usually accompany this triad and may precede the attacks up to 20 minutes.

The vertigenous bouts may last from a few minutes to 2 hours, and their limited duration is characteristic for the disease. Hearing loss usually involves the low frequency spectrum, especially in the early stages of the disease.

However, physicians may not come across the classic full-blown picture of Meniere’s disease from the outset. Various forms of the disease may be encountered in early stages, with symptoms arising either from the vestibular or from the cochlear system. Hearing loss may be very mild at this stage, and patients may actually not at all complain about it, especially when tinnitus and vertigo prevail.

The disease tends to relapse episodically. A full restoration in hearing can be expected to follow an attack of the disease at the initial stages. However, the pattern of hearing loss is fluctuating in the advanced stages and may finally become severe or progressive.

In an effort to reach a consensus regarding Meniere’s definition, the American Academy of Otolaryngology-Head and Neck Surgery (AAOHNS)-Subcommittee of Hearing and Equilibrium and its Measurements classified the diagnosis of the disease into four levels of certainty: certain, definite, probable and possible[5,6] [Table 1]. The “certain” level in the diagnosis is based on post-mortem examination, suggesting that the diagnosis of Meniere’s disease relies on probability rather than certainty. AAOHNS has also classified the severity of hearing loss into four stages. This classification was based on the average pure-tone thresholds at 0.5, 1, 2, and 3 kHz, using the worst audiogram of a 6-month interval before treatment [Table 2].[6]

**Table 1:** Level of certainty regarding Meniere’s disease

Symptoms and findings	Level of certainty			
	Certain	Definite	Probable	Possible
Histopathological findings	+	–	–	–
Rotatory vertigo	++^a^	++	+	+ or –c
Hearing loss	++	++	+	– or +^c^
Tinnitus	++	++	+	–
Aural fullness	+/–b	+/–	+/–	–

a The number of marks represents the minimum number of episodes, not their severity,

b May not be present,

c Symptoms are mutually exclusive

**Table 2:** Stages of Meniere’s disease based on hearing levels

Stage 1: A four-tone average of less than 26 db
Stage 2: A four-tone average between 26 and 40 db
Stage 3: A four-tone average between 41 and 70 db
Stage 4: A four-tone average of more than 70 db

The prevalence of Meniere’s disease in Northern European countries seems markedly high: approximately 430 cases per million are reported in Finland[7] and 460 cases per million in Sweden.[8] A very high prevalence is also encountered in the UK (1000 patients per million), whilst an approximate number of 15 new cases per 100,000 people is diagnosed with the disease every year.[9]

There is no difference with regard to gender,[10,11] and the most common age for the onset of symptoms is the fourth decade of life. The diagnosis of Meniere’s disease in patients with an onset of symptoms after the age of 60 years is very rare. Up to 50% of cases may be bilateral, although the other ear may be affected after several years.[9] The mean conversion time form unilateral to bilateral disease is 7 years.[12]

It is interesting to note that Meniere’s disease seems to have a considerable impact on primary healthcare systems, as it has been estimated that an average GP practice with some 6000 patients would expect to see perhaps one new case per year. A Health Authority of 250,000 patients would see some 60 cases per year. This means, in turn, that the cost of treatment, when set against the cost of not treating patients, who, if left untreated, may have up to 20 attacks of long duration per month, and consume much GP and specialist clinic time, undoubtedly favors the efforts toward successful management of the disease, in terms of cost effectiveness.[13]

### Etiology and pathophysiology of Meniere’s disease

The etiology and pathogenesis of the disease remain elusive. An autosomal dominant pattern with features that may indicate anticipation is suggested in familial cases.[14] Endolymphatic hydrops is accepted as the most possible pathophysiologic mechanism of the disease; however, not all cases with hydrops become clinically apparent. Indeed, Seo *et al*.[15] described three cases without vertigo, involving patients with cochleosaccular endolymphatic hydrops, revealed by furosemide-loading vestibular evoked myogenic potential test.

The endolymphatic hydrops is the result of a dysfunction in the mechanism of production and absorption of the endolymph. Although an overproduction of the endolymph has been proposed, it seems more probable that a defect in the absorptive activity of the endolymphatic duct and sac is present.[16] Many studies indicate a possible role of antidiuretic hormone (ADH) in the pathogenesis of Meniere’s disease, and especially in the mechanism of induction of the endolymphatic hydrops. Lim *et al*.[17] assessed ADH levels in 26 patients and although they did not find statistically higher ADH levels in patients with unilateral Meniere’s disease, they could not exclude this possibility for patients with bilateral Meniere’s disease.

Schuknecht *et al*.[18–20] proposed the theory of small ruptures in the membranous labyrinth, which may cause a sudden mixture between perilymph and endolymph, and result in physical and chemical changes in the cochlear and vestibular system, in order to explain the clinical symptoms from both these systems. The recurrences may very well be attributed to a subsequent rupture, which may follow healing from previous damage and a symptom-free interval. However, ruptures are not seen in all temporal bones with Meniere’s disease, and the entire complex of symptoms cannot be fully explained with this theory.[16]

### Diagnosis

#### History–Physical examination

The diagnosis of Meniere’s disease seems to be very easily given to dizzy patients from various medical specialties (i.e. general practitioners, medics, etc.), before patients are even referred to specialists (ENT surgeons, audio-vestibular physicians, neurologists), and even though they may not actually suffer from this specific disease. However, establishing the diagnosis of Meniere’s disease often takes time because of the nonspecific clinical symptoms in the early stages.

The typical clinical triad of symptoms from the vestibular and cochlear systems is usually the key for clinical diagnosis. The duration of acute rotatory vertigo in Meniere’s disease usually ranges from 20 minutes to 2 hours. It is rare for the bout to last several hours or even more in the affected individuals.

Tinnitus accompanies these episodes and may precede the attacks up to 20 minutes. Aural fullness is also quite characteristic of the disease.

The audiometric results vary and depend upon the stage of the disease. They include a low frequency sensorineural hearing loss, although different patterns may also be seen. Physicians may encounter a conductive hearing loss in very early stages, which may be mistaken as middle ear effusion. A mixed type of hearing loss is also possible in these stages and fluctuation is not rare. However, in later stages, a flat pattern in the audiogram is usually seen and the sensorineural hearing loss may become severe to profound.[21]

#### Diagnostic tests

As there is no pre-mortem test to confirm the presence of endolymphatic hydrops, objective diagnostic testing is very important in order to improve the level of diagnostic certainty. Glycerol dehydration test and electrocochleography (EcoChG) are the main diagnostic tests for Meniere’s disease.

The combination of glycerol dehydration test and audiometry has a high sensitivity in the diagnosis of Meniere’s disease. After a baseline audiogram is performed, the patient takes 100 g of 95% glycerol with the same amount of water per os. Another audiogram is performed 90 minutes and 3 hours after ingestion. The test is considered positive when there is an improvement of 10 db or more in pure-tone thresholds at two or more frequencies, or an improvement of 10% of speech discrimination scores. In addition, improvements in postural control may also be expected, and have actually been observed in as many as 70% of patients, who are undergoing the test during disease attacks.[22]

With regard to the time of glycerol testing, a higher sensitivity at the period of symptom onset, rather than during disease-free intervals, was reported by Zhao *et al*. (83.3% as opposed to 43.1%), thus indicating that the respective testing should be preferably carried out at the earlier stages of the disease.[23] In addition, a fluctuating pattern of hearing loss seems to be associated with more consistent differences in pure-tone thresholds and/or speech discrimination scores, after glycerol administration.[24] According to Lu *et al*.[25] the sensitivity of glycerol testing in the diagnosis of Meniere’s disease does not seem to differ significantly from that of ECochG (53.1 and 54.7%, respectively); a positive predictive value of 66% for glycerol testing has also been reported.[26] A more reliable estimation of the inner ear status can be obtained when distortion-product otoacoustic emissions are measured in addition to conventional audiometric testing.[27] Furthermore, the glycerol test is also quite effective in predicting the progression of atypical Meniere’s disease to more definite disease patterns, when it is combined with ECochG.[28]

**Figure 1:**
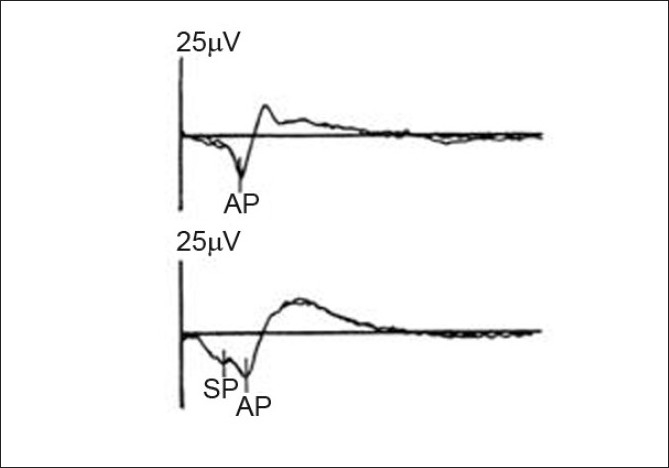
ECoCG tracings SP/AP ratio (upper trace: normal ear, lower ear: hydropic ear) (Seo T, Node M, Miyamoto A, Yukimasa A, Terada T, Sakagami M. Three cases of cochleosaccular endolymphatic hydrops without vertigo revealed by furosemide-loading vestibular evoked myogenic potential test. Otol Neurotol 2003;24:807-11)

ECochG has also been widely used in the diagnosis of Meniere’s disease. During the EcoChG, a needle electrode is placed either through the tympanic membrane on the promontory, or on the tympanic membrane, or simply in the ear canal. The components measured are: a) cochlear microphonics, b) summating potentials (SP), and c) action potentials (AP). The cochlear microphonics and the summating potentials reflect the cochlear bioelectric activity, while the action potentials reflect the activity of distal afferent fibers of the 8th nerve. In ECoChG, we determine the amplitude of the SP and the AP from a common baseline [Figure 1]. The ratio SP\AP is calculated and reported as a percentage. The cut-off criterion for the normal SP/AP amplitude ratio is 50% (0.5) for the ear canal electrode type, 40% (0.4) for the tympanic membrane electrode, and 30% (0.3) for the transtympanic electrode type.[29] An increased level of SP/AP amplitude ratio points to the diagnosis of Meniere’s disease.

The reported sensitivity and specificity of the SP/AP amplitude ratio in the diagnosis of Meniere’s disease vary in the literature. Hall and Antonelli reported a sensitivity of 57% and specificity of 94%,[29] while Chung *et al*. reported a sensitivity of 71% and a specificity of 96%.[30] Devaiah *et al*. reported a sensitivity of about 60%, which reaches 92% when the ECochG is performed during a symptomatic period.[31] By comparing the results of transtympanic and extratympanic ECochG, Ghosh *et al*. reported a difference of 10% in the respective sensitivities and specificities of the SP/AP amplitude ratio, claiming that transtympanic ECochG may have a sensitivity of 100% and a specificity of 90%, depending on the cut-off criterion.[32]

**Figure 2:**
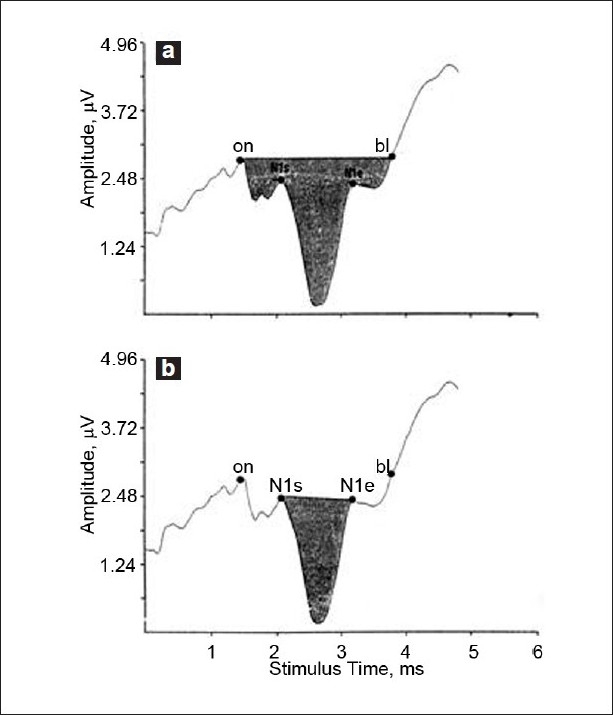
ECoCG tracing for summating potential (SP/AP) area ratios. Stimulus onset (on), start of N1 deflection (N1s), end of N1deflection (N1e) and baseline return (bl) are marked. (a) Software subroutines calculate the ratio of SP area (from on to bl). (b) AP area (from N1s to N1e) is determined by similar subroutines (Devaiah AK, Dawson KL, Ferraro JA, Ator GA. Utility of area curve ratio electrocochleography in early Meniere’s disease. Arch Otolaryngol Head Neck Surg 2003;129:547-51)

Devaiah *et al*. proposed a new component for ECochG with higher sensitivity, especially in early stages.[31] This component is the SP/AP area curve ratio [Figure 2]. The upper normal limit for this component was 1.94 and for the SP/AP amplitude ratio was 0.53. The SP/AP area curve ratio was found abnormal in seven of the eight patients studied (87%), while the SP/AP amplitude ratio was found abnormal in only four patients (50%) with possible Meniere’s disease. This refinement in ECochG analysis may contribute to an earlier diagnosis of Meniere’s disease.

Young *et al*. reported that another examination [vestibular evoked myogenic potentials (VEMPs)] highly correlates with the stages of Meniere’s disease and may be used as another aid to assess the stage of the disease.[33]

When the test is performed within 24 hours of a Meniere attack, VEMPs may be abnormal in up to 67% of patients. However, after 48 hours, half of the patients with initially abnormal VEMPs may return to normal.[34]

However, compared to ECoG, the VEMP test showed a total rate of 58% for positive findings in an 11-year period, which was quite lower than the respective percentage of ECoG (77%).[35] Therefore, further studies to optimize the clinical utility of VEMPs in the diagnosis of Meniere’s disease are warranted.

#### Additional assessment and differential diagnosis

There are many other tests and examinations that may help ENT specialists, audio-vestibular physicians, or neurologists in the diagnosis, and especially in the differential diagnosis of Meniere’s disease. Blood tests such as a full blood count, glucose, cholesterol, and thyroid function tests, erythrocyte sedimentation rate, and autoimmune tests [C-reactive protein, immunoglobulins, total complement, antinuclear factor, and anticochlear antibody (anti-68KD)] may be very helpful in order to exclude a metabolic or an immune-mediated disease, especially when the symptoms and signs are bilateral. A retrospective review of 211 consecutive patients with classic Meniere’s disease suggested that treponemal antigen tests for syphilis are cost-effective in evaluating patients with Meniere’s disease. However, the extensive testing of glucose metabolism and thyroid function is probably not indicated, at least in patients without a history suggestive of a metabolic disorder.[36]

Caloric testing may reveal a unilateral vestibular hypofunction, even though up to 50% of patients with Meniere’s disease may have normal findings. The results of this test are unfortunately nonspecific for Meniere’s disease and may fluctuate over time for a given patient. Moreover, standard calorics only assess the lateral semicircular canal. Nevertheless, caloric testing is also considered cost-effective in evaluating patients with Meniere’s disease.[36]

Auditory brainstem responses (ABR) may discriminate Meniere’s disease from retrocochlear pathologies (such as an acoustic neuroma). This is very important because Meniere’s disease may mimic retrocochlear or even central pathologies. In addition, a recent study reported that high-pass noise masking of ABR allows an undermasked wave V to appear in Meniere patients, at latencies similar to those obtained in the unmasked condition, while data from the normal hearing group demonstrate no undermasked wave V (or significantly delayed in latency), due to the masking noise. The distribution of latency differences between groups reportedly contains no overlap, thus providing 100% sensitivity and 100% specificity, and may be used in the differential diagnosis of Meniere’s disease and in the monitoring of treatment.[37]

High resolution computed tomography may be used to exclude any other pathology, mainly in the middle ear, or to assess the vestibular aqueduct. Magnetic resonance imaging (MRI) should also be used in two distinct occasions:

- When the latency of wave V in the ABR is delayed and there is a possibility of a retrocochlear pathology (i.e. acoustic neuroma) and
- To assess the membranous labyrinth and enhance the level of certainty for the diagnosis of Meniere’s disease.[21]

**Figure 3:**
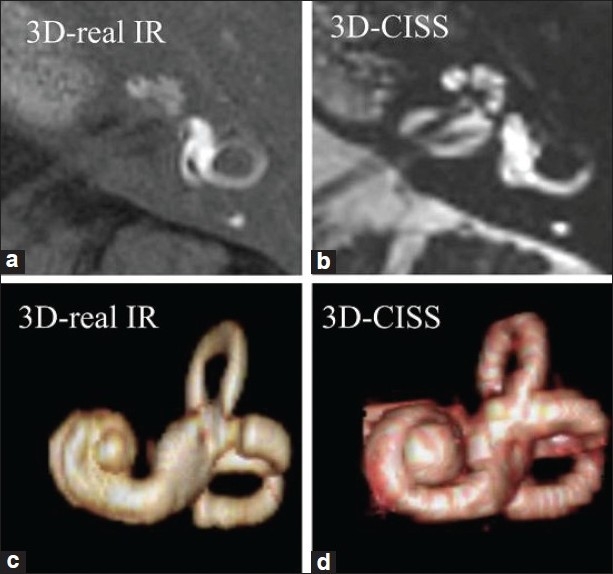
MRI protocols in a patient with Meniere’s disease. High spatial resolution 3D-real inversion recovery (IR) image (a, 0.8 mm thick) and 3D-constructive interference in the steady-state (CISS) image (b, 0.4 mm thick), and their volume-rendered (VR) images (c, d). By comparing the perilymphatic VR image (c) and total lymphatic VR image (d), we can appreciate the degree of endolymphatic hydrops three dimensionally (Naganawa S, Nakashima T. Cutting edge of inner ear MRI. Acta Otolaryngol 2009;129:15-21)

Even though no imagine technique is specific enough to set the diagnosis of Meniere’s disease alone, the recent use of 3D fluid-attenuated inversion recovery (3D-FLAIR) MRI protocols in 3 T field strengths can delineate the perilymphatic and endolymphatic spaces of the inner ear after intratympanic injection of Gadolinium DTPA (Gd-DTPA).[38] Vestibular enhancement is observed first, followed by advance of the enhancement to the basal cochlear turn and semicircular canals, and finally, the apical turn of the cochlea. 24 hours is the optimal interval between Gd administration and MR examination, when evaluating the whole labyrinthine system.[39] In patients with endolymphatic hydrops, the perilymphatic space surrounding the endolymph is either small or cannot be visualized [Figure 3].[38]

Several other tests have also been used in the diagnosis of Meniere’s disease, such as the vestibular autorotation test, the video-occulography, and the traveling-wave velocity technique.[21] However, their value has not been fully assessed, and up to now, they are not routinely used.

In addition, the contralateral ear should also be assessed for the potential presence of an incipient disease in cases of unilateral Meniere’s,[40] as more than 50% of patients with bilateral disease demonstrate involvement of the second ear at least 2 years after symptom onset in the first ear.[41] Elevated antinuclear antibodies are the most notable finding in patients with bilateral Meniere’s disease, compared with their unilateral counterparts.[42]

It should also be mentioned that despite extensive research, none of the aforementioned diagnostic tests has so far demonstrated any clear-cut correlation to a definite benefit from potential surgical interventions. Hence, their role in arriving at the decision to consider surgical treatment is still quite limited. However, extensive vestibular testing, in order to determine the residual labyrinthine function of the contralateral ear, should always be exercised, especially when ablative surgical treatment is considered.[43] ECochG monitoring may also maximize the likelihood of a successful intervention in patients with abnormal baseline measurements.[44]

The differential diagnosis of Meniere’s disease includes otosclerosis, especially the cochlear variant, which may manifest vestibular symptoms in about 25–30% of patients.[45] Acute vestibular labyrinthitis or neuronitis may also mimic the attacks of Meniere’s disease. However, the episodes in the latter case are usually shorter and are also associated with auditory symptoms.

ENT specialists, audio-vestibular physicians and neurologists also need to often differentiate Meniere’s disease from other pathologies which can cause subjective tinnitus. Not only otologic conditions (i.e. presbyacousis, noise-induced hearing loss, Meniere’s disease, otosclerosis, etc.), but also metabolic disorders (hypo-, or hyperthyroidism, hyperlipidemia, zinc and vitamin deficiencies), neurologic problems (head trauma, whiplash injuries, multiple sclerosis, meningitis), drugs (aspirin, nonsteroidal anti-inflammatory medications, aminoglycosides, heavy metals), dental disorders (temporomandibular joint syndrome) and psychological factors (depression, anxiety) may be responsible for causing tinnitus [Table 3].[46]

**Table 3:** Diseases and factors causing tinnitus

Otologic factors	Metabolic	Neurologic	Pharmacologic factors	Dental factors	Psychological factors
Presbyacusis	Hypothyroidism	Head trauma	Aspirin compounds	Temporo-mandibular joint syndrome	Depression
Noise-induced hearing loss	Hyperthyroidism	Whiplash injury	Nonsteroidal antiinflammatory drugs		Anxiety
Meniere’s disease	Hyperlipidemia	Multiple sclerosis	Aminoglycosides		
Otosclerosis	Zinc deficiency	Meningitis effects	Heavy metals		
	Vitamin deficiency		Heterocyclic antidepressant		

## Conclusions

Meniere’s disease represents a persistent and recurrent problem for patients and affects their quality of life, especially during periods of acute symptomatology. The typical clinical triad of symptoms from the vestibular and cochlear systems (recurrent vertigo, fluctuating sensorineural hearing loss and tinnitus) is usually the key for clinical diagnosis, even though differential diagnosis is often difficult. Taking into account the frequent nature of the disease in certain countries, efforts for reliable diagnosis, prompt referral, and successful management are undoubtedly cost-effective for healthcare systems.

## References

[1] Meniere P (1861). Memoire sur des lesions de l’oreille interne donnant lieu a des symptomes de congestion cerebrale apoplectiforme. Gaz Med (Paris).

[2] Cunha F, Settanni FA, Ganança FF (2005). What is the effect of dizziness on the quality of life for patients with Meniere’s disease?. Rev Laryngol Otol Rhinol (Bord).

[3] Anderson JP, Harris JP (2001). Impact of Ménière’s disease on quality of life. Otol Neurotol.

[4] Söderman AC, Bagger-Sjöbäck D, Bergenius J, Langius A (2002). Factors influencing quality of life in patients with Ménière’s disease, identified by a multidimensional approach. Otol Neurotol.

[5] Nikolopoulos T (1999). Meniere’s Disease in the fall of the century. Hearing Int.

[6] (1995). Committee on Hearing and Equilibrium guidelines for the diagnosis and evaluation of therapy in Meniere’s disease. American Academy of Otolaryngology-Head and Neck Foundation, Inc. Otolaryngol Head Neck Surg.

[7] Kotimäki J, Sorri M, Aantaa E, Nuutinen J (1999). Prevalence of Meniere disease in Finland. Laryngoscope.

[8] Stahle J, Stahle C, Arenberg IK (1978). Incidence of Ménière’s disease. Arch Otolaryngol.

[9] Saeed S, Penney S (2004). Diagnosis and management of Meniere’s Disease. ENT News.

[10] Celestino D, Ralli G (1991). Incidence of Menière’s disease in Italy. Am J Otol.

[11] Wladislavosky-Waserman P, Facer GW, Mokri B, Kurland LT (1984). Meniere’s disease: a 30-year epidemiologic and clinical study in Rochester, Mn, 1951-1980. Laryngoscope.

[12] House JW, Doherty JK, Fisher LM, Derebery MJ, Berliner KI (2006). Meniere’s disease: prevalence of contralateral ear involvement. Otol Neurotol.

[13] http://www.bandolier.com.

[14] Frykholm C, Larsen HC, Dahl N, Klar J, Rask-Andersen H, Friberg U (2006). Familial Ménière’s disease in five generations. Otol Neurotol.

[15] Seo T, Node M, Miyamoto A, Yukimasa A, Terada T, Sakagami M (2003). Three cases of cochleosaccular endolymphatic hydrops without vertigo revealed by furosemide-loading vestibular evoked myogenic potential test. Otol Neurotol.

[16] Mancini F, Catalani M, Carru M, Monti B (2002). History of Meniere’s disease and its clinical presentation. Otolaryngol Clin North Am.

[17] Lim JS, Lange ME, Megerian CA (2003). Serum antidiuretic hormone levels in patients with unilateral Meniere’s disease. Laryngoscope.

[18] Schuknecht HF (1993). Pathology of the ear.

[19] Schuknecht HF, Igarashi M, Pfaltz CR (1986). Pathophysiology of Meniere’s disease. Controversial aspects of Meniere’s disease.

[20] Schuknecht HF (1963). Meniere’s disease: a correlation of symptomatology and pathology. Laryngoscope.

[21] de Sousa LC, Piza MR, da Costa SS (2002). Diagnosis of Meniere’s disease: routine and extended tests. Otolaryngol Clin North Am.

[22] Di Girolamo S, Picciotti P, Sergi B, D’Ecclesia A, Di Nardo W (2001). Postural control and glycerol test in Ménière’s disease. Acta Otolaryngol.

[23] Zhao R, Zhu W, Liu H (2005). The control study of glycerol test in different stage of Meniere’s disease patients. Lin Chuang Er Bi Yan Hou Ke Za Zhi.

[24] Snyder JM (1971). Changes in hearing associated with the glycerol test. Arch Otolaryngol.

[25] Lu JZ, Zhang JG, Lai H (2000). The relationship between ECochG and glycerol test in vertigo patients (report of 112 cases). Lin Chuang Er Bi Yan Hou Ke Za Zhi.

[26] Snyder JM (1982). Predictability of the glycerin test in the diagnosis of Ménière’s disease. Clin Otolaryngol Allied Sci.

[27] Sakashita T, Shibata T, Yamane H, Hikawa C (2004). Changes in input/output function of distortion product otoacoustic emissions during the glycerol test in Ménière’s disease. Acta Otolaryngol Suppl.

[28] Kimura H, Aso S, Watanabe Y (2003). Prediction of progression from atypical to definite Ménière’s disease using electrocochleography and glycerol and furosemide tests. Acta Otolaryngol.

[29] Hall JW, Antonelli PJ, Bailey BJ, Jackler RK, Pillsbury HC, Lambert PR (2001). Assessment of peripheral and central auditory function. Head and Neck Surgery-Otolaryngology.

[30] Chung WH, Cho DY, Choi JY, Hong SH (2004). Clinical usefulness of extratympanic electrocochleography in the diagnosis of Ménière’s disease. Otol Neurotol.

[31] Devaiah AK, Dawson KL, Ferraro JA, Ator GA (2003). Utility of area curve ratio electrocochleography in early Meniere disease. Arch Otolaryngol Head Neck Surg.

[32] Ghosh S, Gupta AK, Mann SS (2002). Can electrocochleography in Meniere’s disease be noninvasive?. J Otolaryngol.

[33] Young YH, Huang TW, Cheng PW (2003). Assessing the stage of Meniere’s disease using vestibular evoked myogenic potentials. Arch Otolaryngol Head Neck Surg.

[34] Kuo SW, Yang TH, Young YH (2005). Changes in vestibular evoked myogenic potentials after Meniere attacks. Ann Otol Rhinol Laryngol.

[35] Wu Z, Zhang S, Zhou N, Yi F, Chen A, Xie S (2006). Significance of some otologic function tests in dignosis of Meniere’s disease. Lin Chuang Er Bi Yan Hou Ke Za Zhi.

[36] Meyerhoff WL, Paparella MM, Gudbrandsson FK (1981). Clinical evaluation of Ménière’s disease. Laryngoscope.

[37] Don M, Kwong B, Tanaka C (2005). A diagnostic test for Ménière’s Disease and Cochlear Hydrops: impaired high-pass noise masking of auditory brainstem responses. Otol Neurotol.

[38] Nakashima T, Naganawa S, Sugiura M, Teranishi M, Sone M, Hayashi H (2007). Visualization of endolymphatic hydrops in patients with Meniere’s disease. Laryngoscope.

[39] Naganawa S, Nakashima T (2009). Cutting edge of inner ear MRI. Acta Otolaryngol Suppl.

[40] Salvinelli F, Trivelli M, Greco F, Casale M, Miele A, Lamanna F (2002). Unilateral endolymphatic hydrops: what about the contralateral ear?. Rev Laryngol Otol Rhinol (Bord).

[41] Paparella MM, Griebie MS (1984). Bilaterality of Meniere’s disease. Acta Otolaryngol.

[42] Ruckenstein MJ, Prasthoffer A, Bigelow DC, Von Feldt JM, Kolasinski SL (2002). Immunologic and serologic testing in patients with Ménière’s disease. Otol Neurotol.

[43] Westhofen M (1992). Preoperative vestibular diagnosis in therapy of Menière’s disease. HNO.

[44] Huang TS, Hsu JC, Lee FP (1994). Electrocochleographic monitoring in endolymphatic sac surgery for Menière’s disease. Arch Otolaryngol Head Neck Surg.

[45] Roland PS, Meyerhoff WL, Bailey BJ, Jackler RK, Pillsbury HC, Lambert PR (2001). Otosclerosis. Head and Neck Surgery-Otolaryngology.

[46] Schleuning AJ, Martin WH, Bailey BJ, Jackler RK, Pillsbury HC, Lambert PR (2001). Tinnitus. Head and Neck Surgery-Otolaryngology.

